# The War Cripple: His Restoration, Occupations, and Future

**Published:** 1917-07-28

**Authors:** 


### THE WAR CRIPPLE.

### His Restoration, Occupations, and Future.

Among the practical problems created by the war, and destined to endure for many years after the fighting has ceased, is that of men disabled in such a fashion and to such a degree that, left to themselves, they will be unable to return to their former occupations, or, in not a few instances, even to do any work at all. The position is important for the men themselves and important also for the nation. For the individual it means a life of idleness and hopelessness, with the dangers which these conditions involve, while for the nation there is a corresponding loss of economic values. The men will not be destitute for they will have their pensions, and in this fact there must be to some a temptation and a danger. On the broadest lines, therefore, a claim arises for an endeavour to meet the situation. Now, it is certain that to a very large extent the situation can be met. There are, indeed, sad and terrible cases in which injuries are beyond compensation and repair, and in which nothing remains but efficient medical and nursing care in association with social efforts to brighten and sustain the maimed lives of the sufferers. But, thanks to the ingenuity and skill which have wedded mechanical dexterities to therapeutic ends, it is now happily possible by compensatory and educational methods to overcome defects and hindrances which formerly would have been regarded as beyond succour. Confidence and usefulness, and the self-respect and joy of living which go with these, can thus be restored, and the man who in an earlier day would have been condemned as a hopeless cripple can be re-established in a position of independence and enabled to take his place as one of the nation's workers. The evil exists, but it can largely be redressed. So valuable is the promise that the time, the effort, and the money required will be sure of an abundant reward.

What is true of our own country in this respect is equally true of the other belligerent nations, and it is therefore seemly that the skill and experience of the Allies should be thrown into a common stock, and that upon this wide basis should be organised a definite and common plan of campaign. This procedure has been followed, and as a result of conferences held in Paris there has been produced a series of recommendations which have expert value and authority, and which include a comprehensive survey of the whole field. At the outset there is need for the instruction of surgeons in the therapeutic value of mechanical and educational methods. No doubt, in a general sense, knowledge of this order already exists. But it is wanting both in range and in precision, and it will never get a fair field of application until surgeons are made to realise that many disabilities which may not be removed by the knife can be successfully met by a less dramatic proceeding. There is, perhaps not unnaturally, a disposition to treat mechanical therapeutic methods as a quite inferior branch of the surgical art and one hardly worthy of the attention of the skilled craftsman. But such an attitude is possible only to those who deliberately close their eyes to modern developments. The truth is that by careful study and exact observation mechanical agencies have been so arranged and adjusted that when properly applied and in suitable cases they are capable of producing therapeutic triumphs of high value, and this over a very wide field. The war in this respect has brought multiplied opportunities, and no professional prejudice must interfere with their utilisation. Again, it is necessary to say that the methods of treatment here in question are not to be regarded as suitable only when surgical interference no longer offers any prospect of relief. The victim must not be detained until the classical methods are seen to be useless, and then sent in some vague fashion to be rubbed or exercised or instructed. On the contrary, the skilled and expert physiotherapeutist must have his say in the matter, and must be in a position, equally with the surgeon, to decide both when the time has arrived for the employment of mechanical aids, and what form and character these aids must take. Like his surgical colleague, he must, of course, be a fully-qualified medical practitioner and capable by his expert knowledge of speaking with the authority of a consultant. Mere empiricism, here as elsewhere, may be able to boast its individual triumphs, but only thoroughness of clinical knowledge can direct wisely a scheme of therapeutics. The circumstances of the day make the need for competent experts in this direction a very pressing one, and it is a just demand that more practitioners be induced to pay attention to such work. Not less insistent is the need for properly organised and equipped institutes. Suitable apparatus must be provided, and trained assistants must be secured and directed. The expense will be considerable, but it is justified both by the claim of duty and by the promise of a more than full repayment.

When the individual patients come to be considered there are two distinct though not unrelated aims to be kept in mind. The one is definite therapeutic treatment intended to restore functional activity to parts disabled by such happenings as muscular weakness, stiffness of joints, and the rest. The other is the education, or re-education, of men on whom has been inflicted some permanent and irremovable defect; the purpose here is the re-establishment of the man as a craftsman in spite of a handicap which cannot be removed. Of efforts in both of these directions we hear now and again in the shape of appeals for support. Such efforts command our full sympathy. What we here wish to emphasise is the large situation which has to be met and the co-operative schemes that are being devised to meet it. That the physio-therapeutist has before him a large opportunity is certain, and we cannot doubt that our British *confrères* will prove themselves equal to the occasion. If some of their colleagues know no possibilities outside the operating theatre the fallow ground must be broken up by educational vigour and persistence.

